# Entwicklung und Evaluation komplexer Interventionen in der Pflege: Anwendung des Rahmenmodells des Medical Research Council am Beispiel von Interventionen zur Vermeidung freiheitsentziehender Maßnahmen

**DOI:** 10.1007/s00103-023-03689-1

**Published:** 2023-04-19

**Authors:** Jens Abraham, Ralph Möhler

**Affiliations:** 1 Institut für Gesundheits- und Pflegewissenschaft, Medizinische Fakultät, Martin-Luther-Universität Halle-Wittenberg, Halle (Saale), Deutschland; 2 Institut für Versorgungsforschung und Gesundheitsökonomie, Centre for Health and Society, Medizinische Fakultät und Universitätsklinik Düsseldorf, Heinrich-Heine-Universität Düsseldorf, Moorenstr. 5, 40225 Düsseldorf, Deutschland

**Keywords:** Freiheitsentziehende Maßnahmen, Interventionsentwicklung, Komplexe Interventionen, Krankenhaus, Pflegeheim, Physical restraints, Intervention development, Complex interventions, Hospital, Nursing home

## Abstract

Viele Interventionen in der Pflege sind komplex. Sie bestehen beispielsweise aus verschiedenen Interventionsteilen (Komponenten) und zielen auf Veränderungen von Prozessen oder dem Verhalten von Individuen oder Gruppen ab. Ein Rahmenmodell des britischen Medical Research Council beinhaltet methodische Empfehlungen für die Entwicklung und Evaluation von komplexen Interventionen. Diese narrative Übersichtsarbeit beschreibt die Umsetzung der methodischen Empfehlungen des Rahmenmodells am Beispiel von Interventionen zur Reduktion freiheitsentziehender Maßnahmen, z. B. Bettgitter oder Gurte an Stuhl und Bett, in der Krankenhaus- und der Langzeitpflege. Neben den Merkmalen der komplexen Interventionen werden die Entwicklung und theoretische Fundierung der Interventionen, die Prüfung der Machbarkeit und Wirksamkeit beschrieben.

## Abstract (en)

Many nursing interventions are complex. They comprise different intervention elements (components) and aim to change processes or behaviours of individuals or groups. A framework of the British Medical Research Council comprises methodological recommendations for the development and evaluation of complex interventions. This narrative review describes the framework’s methodological recommendations using an example of interventions to reduce physical restraints in hospital and long-term care settings, such as bedrails or belts in chairs and beds. In addition to the characteristics of the complex interventions, the development and theoretical foundation of the interventions as well as the feasibility test and evaluation is described.

## Einleitung

Durch den demografischen Wandel und den Anstieg der Zahl von Menschen mit chronischen Erkrankungen und Pflegebedarf steigt auch die Bedeutung von (professioneller) Pflege. Professionell Pflegende übernehmen Aufgaben in der Prävention und Gesundheitsförderung, Beratung, Initiierung und Kooperation bei interprofessionellen Behandlungen, bei der Förderung der Selbstständigkeit, des Selbstmanagements und der sozialen Teilhabe von erkrankten bzw. pflegebedürftigen Menschen [1]. Der Gegenstandsbereich der Pflege(wissenschaft) zeigt Gemeinsamkeiten mit der Disziplin „Public Health“, z. B. im Bereich der Gesundheitsförderung und Prävention. Bei der individuellen Pflege stehen zwar die Bedürfnisse und Bedarfe der zu Pflegenden im Mittelpunkt, die Populationsperspektive ist in der Pflege(wissenschaft) aber ebenfalls relevant, z. B. bei der Wirksamkeitsprüfung von Interventionen.

Die Pflege bzw. Versorgung von Menschen mit Pflegebedarf beinhaltet meist die Durchführung von unterschiedlichen Pflegemaßnahmen. Beispielsweise beinhaltet die Mobilisation eines Menschen mit Einschränkungen der Bewegungsfähigkeit neben der Unterstützung beim Aufstehen und Gehen auch die Beratung zu bestimmten Bewegungsmustern, die das Aufstehen erleichtern können, zu geeigneten Hilfsmitteln oder zu Anpassungen des Wohnraums, damit die zu pflegende Person z. B. selbstständig das Badezimmer erreichen kann. Es können darüber hinaus eine Schmerzeinschätzung und die Initiierung einer Schmerzbehandlung nötig sein. Pflegeinterventionen, die eher einen populationsbezogenen Ansatz haben, sind z. B. die Optimierung der Prozesse zur Verbesserung der Händehygiene des medizinischen Personals. Viele Pflegeinterventionen sind also komplex und müssen in ein komplexes System implementiert werden.

Ziel dieser narrativen Übersichtsarbeit ist es, die methodischen Herausforderungen bei der Entwicklung und Evaluation komplexer Interventionen in der Pflege zu beschreiben und am Beispiel von Interventionen zur Vermeidung von freiheitsentziehenden Maßnahmen (FEM) zu erläutern. Als Grundlage dient das Rahmenmodell zur Entwicklung und Evaluation von komplexen Interventionen im Gesundheitswesen des Medical Research Council (MRC), einer Forschungsorganisation im Vereinigten Königreich [2].

## Komplexe Interventionen

Die erste Fassung des Rahmenmodells zur Entwicklung und Evaluation von komplexen Interventionen im Gesundheitswesen wurde im Jahr 2000 im Auftrag des britischen MRC entwickelt [3]. Nach der Veröffentlichung folgte ein reger methodischer Diskurs, der zu einer ersten Aktualisierung des Modells im Jahr 2008 führte [4]. Die zweite Aktualisierung wurde 2021 veröffentlicht [2, 5]. Das Rahmenmodell beinhaltet 4 Phasen sowie verschiedene Kernelemente, die in allen Phasen relevant sind (siehe Infobox).

### Merkmale von komplexen Interventionen

Interventionen werden aufgrund von unterschiedlichen Eigenschaften als komplex bezeichnet. Sie bestehen oft aus unterschiedlichen Interventionsteilen (Komponenten), die miteinander in Beziehung stehen bzw. sich gegenseitig beeinflussen können. Diese Wechselwirkungen zwischen Komponenten sind ein Bestandteil der Intervention, daher müssen komplexe Interventionen im Ganzen betrachtet werden und nicht als eine Kombination von Einzelinterventionen [2, 4].

Komplexe Interventionen zielen häufig darauf ab, Versorgungsprozesse oder -strukturen zu verändern bzw. neu zu implementieren und dazu sind Verhaltensänderungen von Individuen oder Gruppen (z. B. Teams von Krankenhausstationen) nötig. Dies stellt eine weitere Quelle von Komplexität dar ebenso wie die Adressaten der Intervention. Dies sind oft unterschiedliche Berufsgruppen bzw. Organisationseinheiten, aber auch Personen mit Pflegebedarf oder spezifischen Erkrankungen und deren Angehörige [2, 5]. Diese Personen oder Gruppen benötigen (Fach‑)Wissen, Fähigkeiten und die Möglichkeit und Motivation, um die Intervention umzusetzen.

Für die Bewertung des „Erfolgs“ einer komplexen Intervention sind häufig verschiedene Parameter wichtig und nicht immer können diese Parameter direkt bzw. mit validen Instrumenten erhoben werden. Neben klinischen Ergebnisparametern werden häufig auch Prozessmerkmale oder Verhaltensveränderungen als Ergebnisparameter erhoben.

Außerdem müssen (komplexe) Interventionen in die Praxis implementiert werden. Dort wirken sie auf bestehende Strukturen, Prozesse, Personen(gruppen) und Kulturen ein, die wiederum einen Einfluss auf die Intervention haben. Kontext beinhaltet neben Rahmenbedingungen des Gesundheitssystems und der Versorgungsumgebung auch soziokulturelle Bedingungen, Einrichtungskulturen sowie Haltungen und Einstellungen der an der Versorgung beteiligten Personen [6]. Bei der Implementierung von komplexen Interventionen entsteht eine Interaktion zwischen Intervention und Kontext. Es sind oft gewisse Anpassungen der Interventionen an Kontextbedingungen nötig, aber durch die Implementierung der Intervention kommt es auch zu Veränderungen der Kontextbedingungen.

Komplexe Interventionen müssen so gestaltet sein, dass Anpassungen an den Kontext möglich sind, ohne dass die Intervention dabei so verändert wird, dass sie ihre Ziele nicht mehr erfüllen kann. Diese Fähigkeit zur Anpassung wird als *Tailoring* bezeichnet und bezieht sich meist auf formale Aspekte der Intervention oder einzelner Komponenten. Inhaltliche Aspekte können oft nicht oder nur geringfügig angepasst werden, weil die Intervention dann möglicherweise ihre Funktion bzw. ihr Ziel nicht mehr erreichen kann [2, 6, 7]. Wenn eine Intervention beispielsweise eine Fallkonferenz mit Pflegenden, Physiotherapeut*innen und Mitarbeiter*innen des Sozialdienstes beinhaltet, bei der das Entlassmanagement für bestimmte Personen geplant werden soll, ist der genaue Zeitpunkt der Fallkonferenz vermutlich variabel. Die Personengruppen, die daran teilnehmen sollen, können aber vermutlich nicht verändert werden, weil dann möglicherweise eine relevante Gruppe nicht ausreichend einbezogen wird.

### Entwicklung von komplexen Interventionen

In Phase 1 des MRC-Rahmenmodells geht es um die Entwicklung einer komplexen Intervention. Die Entwicklung basiert auf einer Beschreibung des „Problems“, das durch die Intervention adressiert werden soll, und dem Ziel der Intervention („Was soll verändert werden?“). Das Ziel muss so formuliert sein, dass es spezifisch, erreichbar und überprüfbar ist.

Anschließend muss ein Wirkmechanismus identifiziert bzw. entwickelt werden, der geeignet erscheint, um das Ziel der Intervention zu erreichen („Wie kann es verändert werden?“). In der aktuellen Fassung des MRC-Rahmenmodells wird die Erstellung einer Programmtheorie empfohlen. Eine Programmtheorie beschreibt den Wirkmechanismus, also wie und unter welchen Bedingungen eine Intervention ihre Wirkung erreichen soll, sowie die Komponenten der Intervention und wie diese interagieren. Zudem werden die Merkmale des Kontextes reflektiert, die den Wirkmechanismus beeinflussen und umgekehrt, wie dieser Mechanismus den Kontext beeinflussen soll [2].

Um eine komplexe Intervention zu entwickeln, sind also verschiedene Schritte nötig. Zunächst muss der aktuelle Stand des Wissens aufbereitet werden, zum Beispiel zu:

- bereits verfügbaren Interventionen, die ein vergleichbares Problem adressieren und ein vergleichbares Ziel verfolgen und als Grundlage für die Entwicklung dienen können,
- Aspekten der aktuellen Versorgungspraxis (z. B. Bedürfnisse der Zielgruppen oder der beteiligten Berufsgruppen, hemmende oder fördernde Faktoren bzgl. einer veränderten Versorgung),
- Kontextbedingungen mit Relevanz für die Intervention,
- theoretischen Grundlagen in Bezug auf den Wirkmechanismus.

Der aktive Einbezug von Interessenvertretungen ist ebenfalls wichtig. Häufig finden sich keine oder nur wenige Informationen zu den oben genannten Themen in der Literatur bzw. ist deren Übertragbarkeit nur teilweise gegeben. Oder es ist unklar, inwieweit ein Interventionskonzept in der Praxis umsetzbar ist. Der Einbezug von geeigneten Interessenvertretungen ist daher wichtig, um die Annahmen zur Intervention und die Akzeptanz eines Interventionskonzeptes zu explorieren. Auch ökonomische Aspekte sind bei der Entwicklung zu berücksichtigen. Die Modellierung, also die Ableitung und Beschreibung der Interventionskomponenten und ihrer Wechselwirkungen, erfolgt dann auf Basis dieser Erkenntnisse und der daraus entwickelten Programmtheorie [2, 6].

#### Beispiel: Entwicklung einer komplexen Intervention zur Vermeidung von FEM im Akutkrankenhaus

Ein „Versorgungsproblem“ anhand dessen die Entwicklung einer Intervention exemplarisch beschrieben werden soll, ist die Anwendung von FEM im Akutkrankenhaus. Als FEM gilt „jede Handlung oder Prozedur, die eine Person daran hindert, sich an einen Ort oder in eine Position ihrer Wahl zu begeben und/oder den freien Zugang zu ihrem Körper begrenzt durch irgendeine Maßnahme, die direkt am oder in unmittelbarer Nähe des Körpers angebracht ist und nicht durch die Person mühelos kontrolliert oder entfernt werden kann“ [8]. Dies beinhaltet beispielsweise die Verwendung von Bettgittern und Gurtfixierungen der Hände oder des gesamten Körpers.

##### Hintergrund.

FEM werden in Akutkrankenhäusern regelmäßig eingesetzt, meist bei Personen mit einem Delir oder einer demenziellen Erkrankung [9, 10]. Die häufigsten Gründe für die Anwendung von FEM sind die Vermeidung von Stürzen bzw. die Kontrolle von Verhaltensweisen, in denen eine potenzielle Gesundheitsgefährdung für die Patient*innen gesehen wird, wie das Umherlaufen von Menschen mit Demenz mit einem erhöhten Sturzrisiko. Außerdem werden FEM eingesetzt, um Handlungen zu unterbinden, die zum ungeplanten Entfernen von Drainagen, venösen Zugängen oder Nasensonden führen könnten [10–12].

Allerdings sind FEM meist nicht geeignet, um die genannten Ziele zu erreichen, und ihre Anwendung ist mit verschiedenen unerwünschten Wirkungen assoziiert [10]. Beispielsweise verringert die Anwendung von FEM bei unruhigen Patient*innen mit kognitiven Einschränkungen die Unruhe nicht, sondern verstärkt sie häufig noch [13, 14]. Auch der Nutzen von FEM zur Vermeidung von Stürzen ist nicht belegt. In vielen Studien zeigt sich keine relevante Reduktion der Stürze durch die Anwendung von FEM und die Reduktion von FEM führt meist nicht zu einem Anstieg der Stürze. Allerdings ist die Evidenz hier nicht eindeutig und stammt aus Beobachtungsstudien [15, 16]. Der Einsatz von Bettgittern kann sogar die Gefahr von sturzbedingten Verletzungen erhöhen, wenn Patient*innen das Bettgitter übersteigen und aus größerer Höhe stürzen. Auch der Zusammenhang zwischen der Anwendung von FEM und einer Reduktion der Anzahl an ungeplanten Extubationen durch Patient*innen ist nicht eindeutig belegt; die Extubationsraten sind auch bei Patient*innen mit FEM oft hoch [17]. Die Anwendung von FEM ist darüber hinaus mit unerwünschten Effekten assoziiert, wie dem Abbau der körperlichen Funktionsfähigkeit und einer längeren Krankenhausverweildauer [18]. Leitlinien empfehlen daher FEM möglichst nicht bzw. nur als allerletzte Option anzuwenden [11].

##### Ziel und Wirkmechanismus.

Obwohl der mangelnde Nutzen und die negativen Konsequenzen der Maßnahmen in vielen Studien beschrieben wurden und in Leitlinien die Vermeidung von FEM empfohlen wird, halten viele Mitarbeiter*innen im Krankenhaus FEM nach wie vor für geeignete Maßnahmen zur Sturzprophylaxe. So werden bei einem erhöhten Sturzrisiko weiterhin FEM oft anstelle von spezifischen Maßnahmen zur Sturz- oder Verletzungsprophylaxe eingesetzt. Ursachen für die hohen Prävalenzraten von FEM sind daher vermutlich fehlendes Wissen des Personals sowie eher unkritische individuelle oder organisationale Haltungen bzgl. des Einsatzes von FEM [19, 20]. Das Ziel der Intervention war deshalb, die erstmalige Anwendung von FEM bei Patient*innen zu vermeiden bzw. bereits eingesetzte FEM zu reduzieren. Der Wirkmechanismus der Intervention setzte bei der Veränderung der Haltungen der verschiedenen an der Versorgung beteiligten Berufsgruppen und der Einrichtungskultur an. Darüber hinaus sollten Barrieren zur Reduktion oder Vermeidung von FEM überwunden werden, wie das fehlende Wissen zu alternativen Versorgungsstrategien [21]. Da für das Krankenhaussetting keine wirksamen Interventionen vorlagen [22], orientierte sich die Modellierung der Intervention an einer komplexen Intervention, die in Deutschland für die stationäre Langzeitpflege entwickelt wurde und deren Wirksamkeit belegt ist [23].

##### Modellierung der Intervention.

Als theoretische Grundlage für die Intervention wurde die „Theorie des geplanten Verhaltens“ von Ajzen gewählt [24]. Die Theorie beschreibt 3 Faktoren, die das Handeln beeinflussen: (1) die Einstellungen der handelnden Person(en), (2) die sozialen Normen des Umfelds bzgl. des Verhaltens und (3) die wahrgenommene Verhaltenskontrolle (Erwartung dazu, ob ein Verhalten einfach oder schwierig umgesetzt werden kann). Bei der Modellierung der Intervention wurden solche Komponenten ausgewählt, die nach den 3 Faktoren der Theorie geeignet erschienen, eine Verhaltensänderung in der Praxis in Bezug auf die Anwendung von FEM zu erreichen. Als Kernkomponente wurden die Qualifikation und Implementierung von Multiplikator*innen (von den Abteilungsleitungen benannte Pflegende) festgelegt. Diese sollten in ihren Abteilungen die Veränderung der Kultur anstoßen und deren Umsetzung fördern und begleiten (diese Komponente adressierte alle 3 oben genannten Faktoren der Theorie). Der Einsatz von Multiplikator*innen wurde bereits in verschiedenen Studien mit Interventionen zur Verbesserung der Versorgungsqualität erfolgreich umgesetzt [23, 25]. Weitere Komponenten waren eine Kurzschulung für die an der Versorgung beteiligten Berufsgruppen (adressierte die Faktoren 1 und 2), eine formale Unterstützung der ärztlichen und pflegerischen Leitungen (Faktoren 2 und 3) sowie regelmäßige Audit- und Feedback-Runden (Faktoren 2 und 3). Die Intervention sollte nicht nur Pflegende, sondern noch andere Berufsgruppen einbeziehen [21].

### Prüfung der Machbarkeit komplexer Interventionen

Nachdem eine Intervention vollständig entwickelt wurde, soll die Machbarkeit geprüft werden (2. Phase des MRC-Rahmenmodells). Das Ziel dieser Phase ist die Überprüfung von Machbarkeit und Akzeptanz der Intervention (inkl. der Eignung der Implementierungsstrategie) und der Studienprozeduren [2]. Dazu wird die Intervention modellhaft implementiert und eine Pilotstudie durchgeführt.

Im Rahmen der Machbarkeitsprüfung können einzelne Komponenten separat betrachtet werden. Von besonderer Bedeutung sind dabei Komponenten, Prozesse oder andere Aspekte der Intervention, für die in der Entwicklungsphase Unsicherheiten bzgl. der Machbarkeit und Akzeptanz identifiziert wurden (eines der Kernelemente, siehe Infobox). In jedem Fall wird aber die Machbarkeit der Gesamtintervention überprüft. Außerdem kann exploriert werden, ob alle relevanten Kontextfaktoren in der Entwicklungsphase ausreichend berücksichtigt wurden. Wenn die Intervention Studien- oder Implementierungsmaterialien beinhaltet, werden auch diese auf ihre Verständlichkeit und Eignung hin überprüft. Alle relevanten Zielgruppen bzw. Interessenvertretungen sollten einbezogen werden.

Die Machbarkeitsprüfung bzw. Pilotierung der Studienprozeduren bezieht sich auf alle Prozesse, die für die Durchführung einer nachfolgenden Evaluation nötig sind. Dies beinhaltet z. B. Rekrutierungsstrategie und -prozesse und die Datenerhebung.

Die Ergebnisse der Machbarkeitsprüfung können eine Anpassung von Teilen der Intervention, der Gesamtintervention oder von Studienprozeduren oder -prozessen nötig machen. Je nach Umfang dieser Anpassungen kann anschließend eine erneute Machbarkeitsprüfung nötig sein.

In dieser Phase werden meist qualitative und quantitative Methoden eingesetzt, um die unterschiedlichen Aspekte der Machbarkeit zu untersuchen.

#### Beispiel: Machbarkeitsprüfung der Intervention zur Vermeidung von FEM im Akutkrankenhaus

In der oben skizzierten Studie wurde die Machbarkeit der Intervention und der Studienprozeduren mit einem Mixed-Methods-Design überprüft. Eine der während der Entwicklung identifizierten Unsicherheiten war die Umsetzung des interprofessionellen Ansatzes der Intervention. Zielgruppe waren die verschiedenen Berufsgruppen, die an der Entscheidungsfindung zu FEM im Krankenhaus beteiligt sind, wie Pflegende und Ärzt*innen. Auch die jeweiligen Leitungspersonen wurden mit einbezogen.

Die Ergebnisse zeigen, dass der Interventionsansatz grundsätzlich umsetzbar und praktikabel war, die Zahl der teilnehmenden Abteilungen war allerdings gering und nicht alle Komponenten konnten wie geplant umgesetzt werden. Beispielsweise nahmen ausschließlich Pflegende an den verschiedenen Schulungen teil und auch in die Entscheidungsfindung zur Anwendung von FEM konnten keine weiteren Berufsgruppen einbezogen werden, obwohl ein interprofessioneller Ansatz geplant war. Auch die Methode zur Erhebung der Anwendung von FEM über die Routinedokumentation erwies sich als nicht ausreichend valide [21]. Die Intervention wird derzeit in einer Folgestudie weiterentwickelt und an die Bedingungen von weiteren Fachbereichen angepasst. Anschließend wird die Machbarkeit in einer multizentrischen Pilotstudie erneut überprüft und erste Hinweise zur Umsetzung des geplanten Wirkmechanismus, zu Veränderungen bei der Anwendung von FEM sowie Daten für eine Fallzahlberechnung für eine spätere Evaluationsstudie werden erhoben [26].

### Evaluation von komplexen Interventionen

Bei der Evaluation von komplexen Interventionen (3. Phase des MRC-Rahmenmodells) steht oft die Prüfung der Wirksamkeit im Vordergrund. In der zweiten Aktualisierung des MRC-Rahmenmodells werden aber noch weitere Forschungsperspektiven skizziert.

Theoriebasierte Fragestellungen untersuchen den Wirkmechanismus und die Bedeutung von Kontextbedingungen näher. Das Ziel ist es, zu verstehen, wie eine Veränderung zustande kommt und welche Wechselwirkungen es zwischen dem geplanten Wirkmechanismus, der Intervention mit ihren Komponenten und dem Kontext gibt. Auch die Frage danach, unter welchen (Kontext‑)Bedingungen eine Intervention wirksam bzw. nicht wirksam ist, fällt in diese Forschungsperspektive. Die Ergebnisse sind wichtig zur Prüfung und Weiterentwicklung des Wirkmechanismus und der Programmtheorie.

Die „System“-Perspektive fokussiert darauf, wie sich Intervention und Zielsetting gegenseitig beeinflussen. Die Intervention wird hier als „Eingriff“ in ein komplexes System gesehen und der Fokus liegt auf der Interaktion zwischen der Intervention und dem Kontext, also dem Implementierungsprozess und den Prozessen und Veränderungen im Rahmen der Implementierung [2].

Auch bei Studien zur Wirksamkeit von komplexen Interventionen ist neben der quantitativen Evaluation eine begleitende Prozessevaluation zwingend erforderlich, da die Wirksamkeit von komplexen Interventionen maßgeblich vom Grad der Implementierung abhängt. Bei der Prozessevaluation werden also die Implementierungsprozesse und die Umsetzung der Intervention im Zielsetting untersucht. Letztlich ist die Frage inwieweit der geplante Veränderungsprozess umgesetzt wurde. Außerdem werden hemmende und fördernde Faktoren der Implementierung und ungeplante Veränderungen durch die Intervention im Zielsetting erhoben. Auch für die Prozessevaluation werden in der Regel qualitative und quantitative Methoden eingesetzt [2, 27].

Das MRC-Rahmenmodell empfiehlt für die Überprüfung der Wirksamkeit randomisierte kontrollierte Studien (RCTs) als Design mit der höchsten Aussagekraft. Wenn eine komplexe Intervention nach den methodischen Empfehlungen entwickelt und ihre Machbarkeit überprüft wurde, ist die Durchführung solcher Studien auch in der Pflege möglich. Komplexe Interventionen in der Pflege zielen häufig auf Veränderungen auf Organisationsebene ab und werden daher in Studien auf einzelnen Stationen oder der gesamten Einrichtung implementiert, wie zum Beispiel die oben beschriebene Intervention zur Vermeidung von FEM im Akutkrankenhaus oder in der stationären Altenpflege [28]. In diesen Fällen ist die Randomisierung von Stationen oder Wohnbereichen (Clustern) nötig [29]. Eine methodische Herausforderung bei der Analyse im Rahmen der Prozessevaluation sind die Synthese von Prozessdaten, die mit qualitativen und quantitativen Methoden erhoben wurden, sowie die Interpretation der Ergebnisse der Wirksamkeitsprüfung und der Prozessevaluation [27].

#### Beispiel: Evaluation einer Intervention zur Vermeidung von FEM in der stationären Altenpflege

Für die oben beschriebene Intervention in Akutkrankenhäusern wurde bislang keine Wirksamkeitsstudie durchgeführt. Zuvor wurde jedoch eine komplexe Intervention zur Vermeidung von FEM in der stationären Altenpflege entwickelt und evaluiert [23]. Diese Intervention basierte auf einer evidenzbasierten Leitlinie zur Vermeidung von FEM in der stationären Altenpflege und nutzte ebenfalls einen Multiplikator*innen-Ansatz, um eine Pflege möglichst ohne FEM zu implementieren.

Die Wirksamkeit der Intervention wurde in einer clusterrandomisierten Studie mit 36 Pflegeeinrichtungen (jeweils 18 in der Interventions- und Kontrollgruppe) untersucht. In der Interventionsgruppe wurde die komplexe Intervention implementiert, die Kontrollgruppe erhielt schriftliche Informationsmaterialien zur Vermeidung von FEM. Primärer Endpunkt war der Anteil der Bewohner*innen mit mind. einer FEM (unangekündigt erhoben zu 3 verschiedenen Tageszeiten) nach 12 Monaten. In der parallel durchgeführten Prozessevaluation wurden der Grad der Implementierung und die Umsetzung der Intervention sowie Barrieren und fördernde Faktoren in einem Mixed-Methods-Design untersucht.

Nach 6 Monaten zeigte sich eine Reduktion beim Anteil der Bewohner*innen mit mind. einer FEM in der Interventionsgruppe um 6,5 Prozentpunkte (95 % Konfidenzintervall (KI) 0,6 bis 12,4 Prozentpunkte, *n* = 3670) im Vergleich zur Kontrollgruppe. Der Anteil der Bewohner*innen mit mind. einem Sturz, einer sturzbedingten Fraktur oder einer Verordnung von psychotropen Medikamenten unterschied sich nicht zwischen den Gruppen [23]. Bei der Prävalenz von FEM gab es jedoch zum Teil starke Unterschiede zwischen einzelnen Einrichtungen, sowohl zu Beginn als auch am Ende der Studie.

Die Prozessevaluation hat ergeben, dass die Intervention wie geplant implementiert wurde. Fördernde bzw. hemmende Faktoren waren institutionelle Rahmenbedingungen (wie zeitliche Ressourcen der Multiplikator*innen), Wissen und Haltungen der Pflegenden zur Vermeidung von FEM und die Verfügbarkeit von Hilfsmitteln (z. B. sehr niedrigen Betten).

### Implementierung von komplexen Interventionen in die Regelversorgung

Nach erfolgreicher Prüfung der Wirksamkeit unter optimalen Bedingungen („efficacy“) und unter alltagsnahen Bedingungen („effectiveness“) beschreibt die 4. Phase des MRC-Rahmenmodells die Implementierung in die Regelversorgung. Diese Phase ist bislang jedoch nur sehr allgemein beschrieben worden. Der Fokus in dieser Phase liegt auf der Implementierbarkeit unter den Bedingungen der Regelversorgung, der Prüfung von Langzeiteffekten und von unerwünschten Wirkungen der Intervention [2–4].

#### Beispiel: Implementierung der Intervention zur Vermeidung von FEM in der stationären Altenpflege unter alltagsnahen Bedingungen

Die im vorherigen Abschnitt dargestellte Studie zur Evaluation der Intervention untersuchte deren Wirksamkeit unter kontrollierten Bedingungen („efficacy“) der Intervention [23]. In einer Folgestudie wurde die Wirksamkeit unter alltagsnahen Bedingungen („effectiveness“) evaluiert [28]. Eine Implementierung in die Regelversorgung, wie es die 4. Phase des Rahmenmodells vorsieht, wurde jedoch nicht durchgeführt.

Zur Vorbereitung wurde die evidenzbasierte Leitlinie aktualisiert und eine optimierte Fassung der Intervention anhand der Ergebnisse der Prozessevaluation entwickelt. Der Fokus der optimierten Intervention lag stärker auf Rolle und Qualifikation der Multiplikator*innen (z. B. durch ein ergänzendes Train-the-Trainer-Modul) und der Aufwand für die Implementierung der Intervention in die Pflegeeinrichtungen wurde reduziert [28].

Die Folgestudie nutze ebenfalls ein clusterrandomisiertes Design und es nahmen 120 Pflegeheime in 4 Regionen in Deutschland teil. In der 3‑armigen Studie wurden die optimierte Intervention und die aktualisierte Originalintervention mit einer Kontrollgruppe verglichen (diese erhielt die Leitlinie und zugehörige Materialien). Auch in dieser Studie wurde begleitend eine Prozessevaluation (Mixed-Methods-Design) durchgeführt. Um alltagsnahe Bedingungen zu erreichen, wurden keine Einschlusskriterien außer der Teilnahmebereitschaft definiert und der Zeitraum der Nachbeobachtung verlängert. Bereits bei Studienbeginn zeigte sich zwischen den Einrichtungen eine große Variation beim Anteil der Bewohner*innen mit mind. einer FEM (1–59 %). Nach 12 Monaten zeigte sich ein geringer, statistisch nicht signifikanter Unterschied zwischen den Interventionsgruppen und der Kontrollgruppe (Originalprogramm vs. Kontrollgruppe: −2,0 Prozentpunkte (97,5 % KI: −5,8 bis 1,9); optimierte Intervention vs. Kontrollgruppe: −2,5 Prozentpunkte (97,5 % KI: −6,4 bis 1,4; *n* = 8841, Bonferroni-Korrektur für multiples Testen)). Auch bei Studienende fanden sich weiterhin ausgeprägte Unterschiede bei der Prävalenz von FEM (0–64 %) zwischen verschiedenen Pflegeheimen.

Die Ergebnisse der Prozessevaluation zeigten einerseits, dass die Intervention zwar überwiegend wie geplant implementiert wurde, die Umsetzung des Veränderungsprozesses hin zu einer Pflege möglichst ohne FEM nicht in allen Clustern der Interventionsgruppen im geplanten Ausmaß erreicht wurde. Wichtige Barrieren waren die Haltungen vieler Pflegekräfte, die FEM weiterhin als geeignete Maßnahme zur Sturzprophylaxe ansahen. Auch gab es Hinweise, dass es nur zum Teil gelungen war, die Einrichtungskultur bzgl. der Anwendung von FEM zu verändern. Die Wirksamkeit der Interventionen unter alltagsnahen Bedingungen konnte nicht belegt werden [28, 30]. Es besteht weiterer Forschungsbedarf zu Merkmalen von Einrichtungen, die eine Veränderung der Pflegekultur bezüglich der Anwendung von FEM beeinflussen können, um den Interventionsansatz weiter zu optimieren.

### Berichterstattung zu komplexen Interventionen

In den verschiedenen Phasen der Entwicklung und Evaluation von komplexen Interventionen werden viele Entscheidungen getroffen, die für eine erfolgreiche Implementierung der Intervention in die Praxis oder die Übertragung in andere Kontexte wichtig sind. Daher müssen diese Informationen umfassend und transparent verfügbar sein.

Zur Verbesserung der Berichterstattung wurden Kriterienkataloge (Reporting Guidelines) entwickelt, in denen alle für eine transparente Beschreibung nötigen Informationen enthalten sind. Neben Reporting Guidelines für spezifische Studiendesigns, wie das CONSORT-Statement für RCTs [31], liegen auch entsprechende Kriterienlisten für die Entwicklung und Evaluation von komplexen Interventionen (CReDECI 2; [32]) und zur detaillierten Beschreibung der Interventionsmerkmale (TIDieR; [33]) vor.

## Fazit

Bei der Entwicklung und Evaluation von komplexen Interventionen in der Pflege müssen verschiedene methodische Herausforderungen berücksichtigt werden, die für simple Interventionen weniger relevant sind. Das MRC-Rahmenmodell stellt eine hilfreiche Ressource zur Planung und Strukturierung des Prozesses dar und wurde in der Pflegewissenschaft bereits häufig verwendet [21, 23, 28, 34]. Die aktuelle Fassung hebt die Bedeutung des Wirkmechanismus und der dazugehörigen Programmtheorie sowie des Einbezugs des Kontextes und der relevanten Interessenvertretungen hervor. Auch werden die frühe Planung und Prüfung der Implementierbarkeit der Intervention nun in allen Phasen stärker hervorgehoben und damit wird die Verbindung zwischen Versorgungs- und Implementierungsforschung deutlich.

Herausforderungen bei der Umsetzung der Empfehlungen des Rahmenmodells in der Pflege ergeben sich unter anderem beim Einbezug von Menschen mit Pflegebedarf bzw. kognitiven Einschränkungen. Die aktive Einbindung dieser Gruppen ist aus methodischer und ethischer Perspektive herausfordernd, aber dringend nötig [35, 36]. Zwar können Interessenvertretungen für diese Gruppen in den Forschungsprozess eingebunden werden, inwieweit diese jedoch die Perspektive der Betroffenen ausreichend repräsentieren können, ist nicht geklärt. Es besteht daher Forschungsbedarf zur Stärkung des direkten Einbezugs dieser Gruppen bei der Entwicklung und Evaluation von komplexen Interventionen in der Pflege.

### Infobox

Rahmenmodell des Medical Research Council (MRC) zur Entwicklung und Evaluation von komplexen Interventionen [2].

Das Rahmenmodell beinhaltet 4 Phasen:

1. Entwicklung einer Intervention bzw. Anpassung einer bestehenden Intervention,
2. Prüfung der Machbarkeit,
3. Evaluation,
4. Implementierung in die Regelversorgung.

In der aktuellen Fassung des Rahmenmodells wurden darüber hinaus Kernelemente ergänzt, die in allen Phasen relevant sind:

- Kontext berücksichtigen,
- Entwicklung, Verfeinerung und (erneute) Prüfung der Programmtheorie,
- Interessengruppen einbeziehen,
- wichtigste Unsicherheiten identifizieren,
- Intervention verfeinern,
- ökonomische Überlegungen.
